# ***Alburnoides manyasensis*** (Actinopterygii, Cyprinidae), a new species of cyprinid fish from Manyas Lake basin, Turkey

**DOI:** 10.3897/zookeys.276.4107

**Published:** 2013-03-08

**Authors:** Davut Turan, F. Güler Ekmekçi, Cüneyt Kaya, S. Serkan Güçlü

**Affiliations:** 1 Recep Tayyip Erdogan University, Faculty of Fisheries and Aquatic Sciences, 53100 Rize, Turkey; 2 Department of Biology, Faculty of Sciences, Hacettepe University, Beytepe Campus, 06800 Ankara, Turkey; 3 Süleyman Demirel University, Faculty of Fisheries and Aquatic Sciences, Isparta, Turkey

**Keywords:** Anatolia, Cyprinidae, taxonomy, *Alburnoides*, new species

## Abstract

*Alburnoides manyasensis*, **sp. n.**, is described from the Koca Stream (Lake Manyas drainage, Marmara Sea basin) in Anatolia. It is distinguished from all species of *Alburnoides* in Turkey and adjacent regions, *Alburnoides tzanevi* (Rezovska [Rezve], Istranca and Terkos streams in the western Black Sea drainage), *Alburnoides* cf. *smyrnae* (Banaz Stream, a drainage of Büyük Menderes River, Aegean Sea basin), *Alburnoides fasciatus* (streams and rivers in the eastern Black Sea drainage) and *Alburnoides eichwaldii* (Kura and Aras rivers [a drainage of Kura River], Caspian Sea basin) by a combination of the following characters (none unique to the species):marked hump at nape, especially in specimens larger than 60 mm SL; partly developed ventral keel between pelvic fin and anal fin, scaleless 1/2 to 2/3 its length; body depth at dorsal-fin origin 29−32% SL; caudal peduncle depth 11−12% SL; 45–52+ 2–3 lateral-line scales; 9–12 scale rows between lateral line and dorsal-fin origin; 4–5 scale rows between lateral line and anal-fin origin, 10½–12½ branched anal-fin rays; 40–42 total vertebrae.

## Introduction

The genus *Alburnoides* is characterized by small black spots near the pores located on each side of the lateral line outlining the canal at least along its anterior portion (Bogutskaya and Coad 2009). Three species of *Alburnoides* had been recognized as valid species before 2007. These were *Alburnoides oblongus* Bulgakov, 1923 [Chirchik River, Aral Sea basin], *Alburnoides taeniatus* (Kessler, 1874) [Tashkent, Aral Sea basin], and *Alburnoides bipunctatus* (Bloch, 1782) [France through Europe north of the Alps eastwards to the Black, Caspian and Aral Sea basins] (Berg 1949; Bogutskaya and Naseka 2004). Furthermore, 10 subspecies and local forms were described or reported within the *Alburnoides bipunctatus* complex: *Alburnoides bipunctatus armeniensis* Dadikyan, 1972 [Aras River, Kura River Drainage, Caspian Sea basin], *Alburnoides bipunctatus eichwaldii* De Filippi, 1863 [Kura River, Caspian Sea basin], *Alburnoides bipunctatus rossicus* Berg, 1924 [Dnieper River, Black Sea basin and Volga River, Caspian Sea basin], *Alburnoides bipunctatus rossicus* natio *kubanicus* Berg, 1932 [Kuban River, Sea of Azov basin], *Alburnoides bipunctatus ohridanus* Karaman, 1928 [Lake Ohrid, Adriatic Sea basin], *Alburnoides bipunctatus* var. *prespensis* Karaman, 1924 [Lake Prespa], *Alburnoides bipunctatus* var. *smyrnae* Pellegrin, 1927 [Melel Stream (near İzmir), Aegean Sea basin], *Alburnoides bipunctatus strymonicus* Chichkoff, 1940 [Struma River, Aegean Sea Basin], *Alburnoides bipunctatus tzanevi* Chichkoff, 1933 [Rezovska River (ancient Riesova River as mentioned by Chichkoff and Rezve in Turkish)], Black Sea basin], and *Alburnoides bipunctatus* subsp. (Berg 1949) [Kuma, Terek, Sulak rivers, Caspian Sea basin] (Berg 1949; Bogutskaya and Coad 2009). Besides above mentioned subspecies and local forms of *Alburnoides bipunctatus*, *Aspius fasciatus* Nordmann, 1840 [rivers of the western Transcaucasia, Black Sea basin] and *Alburnoides maculatus* Kessler, 1859 [Salgir River, Sea of Azov basin] have also been synonymized as *Alburnoides bipunctatus*.

Kottelat and Freyhof (2007) reported *Alburnoides bipunctatus ohridanus* and *Alburnoides bipunctatus* var. *prespensis* as valid species. Later, *Alburnoides bipunctatus rossicus*, *Alburnoides bipunctatus rossicus* natio *kubanicus, A. bipunctatus fasciatus, Alburnoides maculatus* and *Alburnoides bipunctatus eichwaldii* were reported as a valid species by Coad and Bogutskaya (2009) and Bogutskaya and Coad (2009). In addition to these, following 10 new species were described: *Alburnoides devolli* Bogutskaya, Zupančič et Naseka, 2010 [Seman River system, Adriatic Sea basin], *Alburnoides fangfangae* Bogutskaya, Zupančič et Naseka, 2010 [Seman River system,Adriatic Sea basin], *Alburnoides gmelini* Bogutskaya et Coad, 2009 [Sunzha River, Terek River drainage, Caspian Sea basin], *Alburnoides holciki* Coad et Bogutskaya, 2012 [Hari (Tedzhen) River, endorheic (historically Aral Sea basin)], *Alburnoides idignensis* Bogutskaya et Coad, 2009 [Bid Sorkh River, Gav Masiab River system, Tigris River drainage, Persian Gulf basin], *Alburnoides namaki* Bogutskaya et Coad, 2009 [Qanat at Taveh, Namak Lake basin], *Alburnoides nicolausi* Bogutskaya et Coad, 2009 [Simareh River in Karkheh River system, Tigris River drainage, Persian Gulf basin], *Alburnoides petrubanarescui* Bogutskaya et Coad, 2009 [Qasemlou Chay, Orumiyeh Lake basin], *Alburnoides qanati* Coad et Bogutskaya, 2009 [qanat in the Pulvar River drainage, Kor River basin], and *Alburnoides varentsovi* Bogutskaya et Coad, 2009 [Ashkhabadka River, endorheic (historically Aral Sea basin)] (Coad and Bogutskaya 2009, 2012; Bogutskaya and Coad 2009, Bogutskaya et al. 2010).

The genus *Alburnoides* is widely distributed in Turkey in rivers and streams of basins of the Marmara, Black and Aegean seas from tributaries of Büyük Menderes River in the west, Euphrates and Tigris river drainages in the east and south-east, and Kura River drainage in the east, being absent only from the Mediterranean Sea basin (Kuru 1975, Erk’akan 1983, Kutrup 1994, Turan 2003, Kuru 2004, Geldiay and Balık 2009, our data). The populations of *Alburnoides* from streams and rivers in the eastern Black sea coast of Turkey were identified as *Alburnoides fasciatus*, and populations from Kura as *Alburnoides eichwaldii* (Bogutskaya et al. 2010).

**Figure 1.**
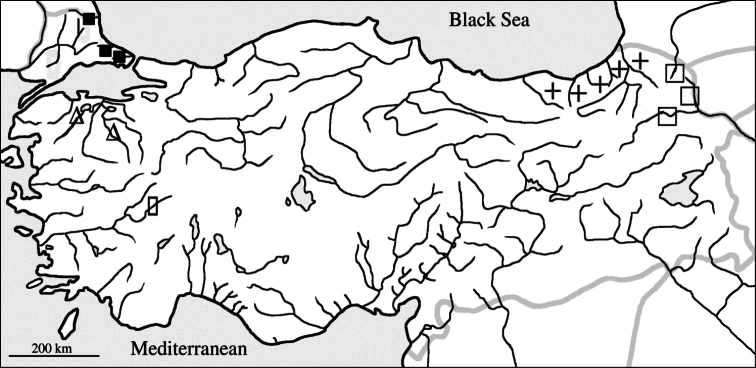
Distribution of named *Alburnoides* species in Turkey: *Alburnoides manyasensis* (∆), *Alburnoides* cf. *smyrnae* ( █ ) *Alburnoides tzanevi* (■), *Alburnoides fasciatus* (+), and *Alburnoides eichwaldii* (□).

In order to understand the *Alburnoides* diversity in Turkey, we sampled at 105 localities throughout the country between 2004 and 2012. In this paper we shall only discuss *Alburnoides* distributed in the Manyas Lake drainage in Marmara sea basin of Turkey (Fig. 1). After comparison with the other *Alburnoides* speciesof Turkey, including Kura River (Caspian Sea basin), Menderes River (Aegen Sea basin), also rivers Terkos, Istranca, Çoruh and İyidere (Black Sea basin), we concluded it is a distinct unnamed species which we describe herein as *Alburnoides manyasensis*.

## Materials and methods

**Figure 2.**
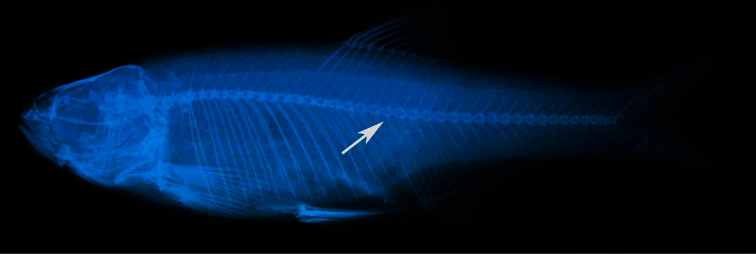
*Alburnoides manyasensis* sp. n. Radiograph of a paratype, FFR 01073, 74 mm SL.Arrow shows first caudal vertebra.

Fish were caught by pulsed DC electrofishing equipment and killed by over overane-stization, fixed in formaldehyde and stored in ethanol. Material is deposited in: FFR, Recep Tayyip Erdoğan University Zoology Museum of the Faculty of Fisheries (Former Fisheries Faculty of the city of Rize). Counts and measurements follow Hubbs and Lagler (1947) except as follows. Head width_1_: the distance between the anterior eye margins; head width_2_: the distance between the posterior eye margins; head width_3_: the head width at the nape; head depth_1_: the head depth through the eye; head depth_2_: the head depth at the nape; snout width: measured at level of the nostrils. The lateral-line scales are counted from the anteriormost scale (the first one to touch the shoulder girdle) to the end of the hypural complex. The scales on the caudal fin itself are indicated by ‘+’. The last two branched dorsal and anal rays articulating on a single pterygiophore are counted as 1½. The number in parentheses after a count indicates the frequency of occurrence of the count. Vertebral counts were obtained from radiographs and counted as total, predorsal, abdominal and caudal vertebrae following Bogutskaya and Coad (2009). Predorsal vertebrae include the Weberian vertebrae and abdominal vertebrae anterior to the first dorsal-fin pterygiophore. Abdominal vertebrae were counted from the first Weberian vertebra to the one just anterior the first caudal vertebra. The first caudal vertebra is that with its haemal spine fully developed (Fig. 2). The count of total and caudal vertebrae includes the last complex vertebra bearing hypurals.

The morphometric characters of the five species of *Alburnoides* from Turkey were compared by Principal Component Analysis (PCA) using a covariance matrix on log–transformed measurements and counts with the software package PAST version 1.8 (Hammer et al. 2001).

## Results

### Alburnoides manyasensis sp. n.

urn:lsid:zoobank.org:act:9A720EDC-D057-4EBB-949C-C298544FE46A

http://species-id.net/wiki/Alburnoides_manyasensis

Fig. 2 3

#### Holotype.

FFR 01069. Female. 82 mm SL; Turkey, Balıkesir Prov., Koca Stream at outlet of Manyas Dam Lake, Lake Manyas drainage; 39°59'26"N, 27°47'58"E, 11 July 2007, coll. D. Turan and R. Buyurucu.

**Paratypes.** FFR 01073, 24, 54–92 mm SL; same data as holotype.

#### Diagnosis.

**Figure 3.**
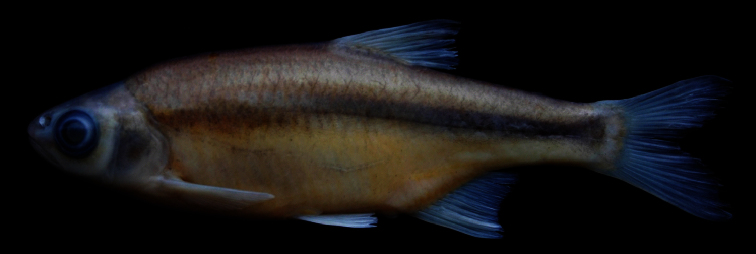
*Alburnoides manyasensis*; Turkey: Balıkesir Province: Koca Stream, Lake Manyas drainage, holotype, FFR 01069, female, 82 mm SL.

*Alburnoides manyasensis* is distinguished from all the species of *Alburnoides* in Turkey and adjacent areas by a combination of the following characters (none unique to the species):partly or slightly developed ventral keel between pelvic fin and anal fin, scaleless about 1/2 to 2/3 its length; upper body profile markedly convex, with marked hump at nape, especially in specimens larger than 60 mm SL; upper head profile straight or slightly convex in interorbital area, slightly convex on snout; corner of mouth reaching vertical through anterior margin of pupil; snout with slightly pointed tip; interorbital width 7–8% SL; dark grey stripe indistinct or slightly distinct in anterior part of body but distinct in posterior body; pigmentation of lateral line slightly distinct in anterior part of body but indistinct in posterior part of body (Fig. 3) in most of specimens; 45–52+2–3 lateral-line scales, 9–12 scale rows between lateral line and dorsal-fin origin, 4–5 scale rows between lateral line and anal-fin origin, 10½–12½ branched anal-fin rays; pharyngeal teeth 4.2–2.4, markedly hooked; total vertebrae 40–42: 20–22, with mode of 21, abdominal and 19–21, with mode of 20, caudal vertebrae.

#### Description.

Maximum known size 92 mm SL. General appearance shown in Fig. 3; morphometric and meristic data given in Tables 1 and 3. Body deep, its depth at dorsal-fin origin 29–32% SL, mean 29.4, at anal-fin origin 22–27% SL, mean 24.4, and markedly compressed. Dorsal profile markedly convex with marked hump at nape in specimens larger than 60 mm SL, ventral profile less convex than dorsal profile. Predorsal length 52–58% SL, mean 54.2. Caudal peduncle deep, its depth 11–12% SL, mean 11.5. Head short, its length 25–27% SL, mean 26.0, approximately 0.8–0.9 times body depth at dorsal fin origin, dorsal profile straight or slightly convex at interorbital area, slightly convex at snout. Snout short, slightly pointed, its length 6–8% SL, mean 6.8, shorter than both eye diameter (eye diameter 7–9% SL, mean 8.2) and interorbital width (interorbital width 7–8 %SL, mean 7.7). Mouth terminal, with very slightly marked chin, its corner reaching vertical through anterior margin of pupil.

Lateral line with 45–52 + 2–3 scales; (9)10–12 scales rows between lateral line and dorsal-fin origin; 4–5 scales between lateral line and anal-fin origin. Gill rakers 8−10: 2–3 + 6–7 on outer side of first gill arch. Dorsal fin with 3 simple and 8½, rarely 7½ or 9½, branched rays; its depth 21–27% SL, outer margin straight or slightly concave. Pectoral fin long, not reaching pelvic-fin origin in both sexes, its length 20–24% SL, outer margin slightly convex, with 12–13 branched rays. Pelvic fin rounded, reaching or slightly behind anus, with 1 simple and 7 branched rays. Anal fin with 3 simple and 10½–12½ branched rays, outer margin concave. Caudal fin forked, lobes slightly pointed.

Pharyngeal teeth 4.2–2.4, markedly hooked. Total vertebrae 40–42; predorsal vertebrae 13–15 with mode of 14; number of abdominal vertebrae 20–22 with mode of 21, and that of caudal vertebrae 19–21 with mode of 20 (frequency of occurrence of character states given in Table 3). Abdominal region longer than caudal region, rarely regions equal, and difference between abdominal and caudal counts varying from 2 to –1; most common vertebral formula 21+20.

**Sexual dimorphism.** There are small tubercles on rays of anal fins in male. The length of the paired fins does not display any statistically significant difference in males and females as it often occurs in other *Alburnoides* species.

**Coloration.** Formalin preserved adults and juveniles brownish on back and upper part of flank, yellowish on lower part of flank and belly. Caudal and dorsal fins light grey; pectoral, pelvic and anal fins yellowish. Spots along lateral line above and below pores slightly distinct in anterior part of body but indistinct in posterior part. Dark grey stripe (its width approximately equal to eye diameter) on upper part of flank from posterior margin of operculum to caudal peduncle, slightly distinct in anterior body part but clearly distinct in posterior part. No or few dark pigment dots on each scale pocket below lateral stripe.

#### Distribution and notes on biology.

*Alburnoides manyasensis* is known only from the Koca Stream, drainage of Lake Manyas, Marmara Sea basin (Fig. 1).It inhabits clear fast running water with cobble and pebble substrates. *Capoeta tinca* (Heckel, 1843), *Barbus oligolepis* Battalgil, 1941,* Squalius cii* (Richardson, 1857),* Vimba vimba* (Linnaeus, 1758),* Chondrostoma* sp.,* Rhodeus amarus* (Bloch, 1782), *Oxynoemacheilus* sp. and *Alburnus* sp.were collected together with *Alburnoides manyasensis*.

#### Etymology.

The name of the species, an adjective, is derived from the name of Lake Manyas.

**Table 1.** Morphometric characters in *Alburnoides manyasensis*, *Alburnoides*. cf. *smyrnae* and *Alburnoides tzanevi*. Mean values are given in parentheses.

	*Alburnoides manyasensis*, n=24			*Alburnoides* cf. *smyrnae*, n=8		*Alburnoides tzanevi*, n=10		*Alburnoides tzanevi*, n=10	
Basin	Marmara Sea			Aegean Sea		Black Sea		Black Sea	
River or stream	Koca			Büyük Menderes		Terkos		Istranca	
	Range	SD	*Holo-type*	Range	SD	Range	SD	Range	SD
Standard length (mm)	54–92		80	58–77		63–81		63–94	
**In percents of standard length**									
Head length	24.5–27.3 (26.0)	0.68	25.2	25.7–27.8 (26.7)	0.71	26.0–27.7 (26.6)	0.58	24.8–26.8 (25.6)	0.71
Body depth at dorsal fin origin	28.8–31.9 (29.4)	1.14	30.0	27.8–29.5 (28.6)	0.64	24.5–27.2 (25.9)	0.95	23.7–26.0 (24.9)	0.83
Body depth at anal fin origin	22.3–26.8 (24.4)	1.26	25.5	23.2–25.2 (24.3)	0.64	21.0–22.9 (22.0)	0.62	20.4–21.8 (21.0)	0.52
Caudal peduncle depth	10.7–12.2 (11.5)	0.42	11.8	10.8–12.3 (11.8)	0.47	10.2–11.0 (10.5)	0.24	8.8–10.3 (9.4)	0.49
Predorsal length	52.4–57.8 (54.2)	1.31	52.7	50.7–55.1 (53.0)	1.29	52.9–54.3 (53.6)	0.48	51.0–53.9 (52.5)	1.00
Prepelvic length	44.4–49.7 (46.8)	1.33	46.9	46.0–47.9 (46.8)	0.71	46.1–48.9 (47.5)	0.87	44.8–48.0 (46.2)	1.23
Preanal length	61.4–67.2 (64.5)	1.54	66.4	63.2–67.7 (65.2)	1.34	64.0–68.8 (66.8)	1.45	65.4–68.0 (66.5)	0.99
Pectoral-fin origin to anal fin	38.1–44.2 (41.3)	1.36	44.2	38.3–42.9 (40.8)	1.73	38.4–44.5 (42.7)	1.87	40.2–44.3 (42.5)	1.38
Pectoral-fin origin to pelvic fin	20.5–24.5 (22.9)	0.94	23.7	19.3–25.0 (22.2)	1.90	21.3–24.6 (22.9)	1.11	20.1–24.4 (22.1)	1.34
Pelvic-fin origin to anal fin	16.7–21.2 (18.6)	1.14	21.2	17.1–20.3 (18.7)	1.10	18.0–21.9 (20.2)	1.16	20.4–22.8 (21.3)	0.83
Caudal peduncle length	20.4–25.0 (22.2)	1.20	22.3	20.7–26.2 (23.7)	2.31	18.2–21.0 (19.5)	1.02	18.1–23.1 (20.4)	1.53
Dorsal fin depth	21.3–27.3 (24.4)	1.64	23.3	21.7–27.9 (24.6)	1.94	20.6–23.4 (21.8)	0.99	20.7–25.6 (22.0)	1.38
Pectoral fin length	20.1–23.6 (21.3)	0.83	20.7	18.6–23.6 (20.6)	2.09	19.2–21.7 (20.2)	0.91	18.4–21.7 (19.9)	0.99
Pelvic fin length	14.5–18.5 (16.8)	0.80	16.5	17.3–21.0 (19.2)	1.18	13.5–17.0 (15.9)	0.99	15.7–17.1 (16.5)	0.47
Anal fin length	15.9–20.9 (18.7)	1.98	18.0	18.4–21.3 (20.0)	1.03	16.9–18.4 (17.5)	0.41	17.3–19.0 (18.1)	0.52
Upper caudal-fin lobe	21.2–27.2 (24.5)	1.73	23.9	27.9–31.3 (29.1)	1.07	21.5–24.4 (22.9)	0.97	22.4–26.8 (23.1)	1.54
Middle caudal-fin lobe	12–15.9 (14.4)	0.99	14.7	14.3–17.3 (16.0)	1.09	13.3–14.7 (14.1)	0.54	12.4 –14.5 (13.4)	0.90
Head width_1_	7.6–9.7 (8.8)	0.64	9.5	8.9–10.3 (9.9)	0.49	8.5–9.7 (9.2)	0.39	7.9–9.6 (9.1)	0.44
Head width_2_	11.5–13.6 (12.7)	0.54	12.8	13.3–15.3 (14.0)	0.58	11.2–12.5 (11.9)	0.44	11.6–12.4 (12.1)	0.32
Head width_3_	12.4–15.9 (13.6)	0.70	13.8	14.2–17.7 (15.0)	0.52	12.2–13.2 (12.8)	0.35	12.4–14.6 (13.1)	0.60
Head depth_1_	13.2–15.0 (14.1)	0.56	14.4	14.4–16.0 (15.2)	0.53	13.8–14.9 (14.3)	0.37	12.3–14.5 (13.6)	0.66
Head depth_2_	18.0–20.4 (19.2)	0.80	19.2	19.8–20.8 (20.3)	0.42	18.0–19.1 (18.7)	0.28	16.5–18.8 (17.5)	0.73
Eye diameter	7.3– 9.1 (8.2)	0.58	7.6	8.1–9.3 (8.7)	0.48	7.0–7.7 (7.4)	0.24	6.6–8.9 (7.7)	0.71
Snout length	6.0–7.7 (6.8)	0.49	6.2	6.4–8.0 (7.0)	0.49	6.4–7.3 (6.8)	0.24	6.3–7.6 (6.9)	0.42
Interorbital width	7.1–8.4 (7.7)	0.36	8.0	8.4–9.5 (8.9)	0.39	6.9–8.0 (7.3)	0.42	6.3–7.5 (6.9)	0.33
Snout width at nostrils	6.8–9.8 (8.3)	0.96	9.4	7.8–9.9 (8.6)	0.68	9.2–10.3 (9.6)	0.36	8.7–10.7 (9.4)	0.35

## Discussion

We compared the material from the Koca Stream in the Marmara Sea basin with *Alburnoides fasciatus* from streams and rivers in the eastern Black Sea basin, *Alburnoides eichwaldii* from Kura and Aras rivers (Kura River drainage) and *Alburnoides tzanevi* from Rezovska (Rezve), Istranca and Terkos streams in the western Black Sea basin.

**Figure 4.**
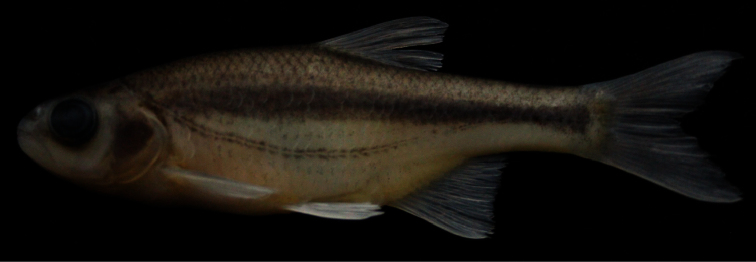
*Alburnoides* cf. *smyrnae***;** Turkey: Uşak Province: Banaz Stream, Büyük Menderes River drainage, FFR 1110, female, 75 mm SL.

No specimens were collected from the type locality of *Alburnoides bipunctatus* var. *smyrnae* although we checked it (Melel Stream, Aegean Sea basin) as well as some streams and rivers near İzmir (ancient Smyrna, Aegean Sea basin, Turkey). Thus, we failed to catch any specimens from the population which is known to exist quite sparsely in the province of İzmir where the habitat is heavily degraded. Instead, we examined 8 specimens of *Alburnoides* from Banaz Stream, a tributary of Büyük Menderes River, Aegean Sea basin. The Banaz Stream population exibitscharacter states similar to those known in *Alburnoides bipunctatus* var. *smyrnae* such as the numbers of lateral-line scales and anal-fin branched rays. They have 41–46 total lateral line scales according to Pellegrin (1928: 86) and 42–47 in our material, and 13–15 branched anal–fin rays according to Pellegrin (1928: 86) and 12½–14½ in our material. The population from the Büyük Menderes River is most probably conspecific with *Alburnoides bipunctatus* var. *smyrnae*. Therefore we identify them as *Alburnoides* cf. *smyrnae* (Fig. 4) and suppose it is a distinct valid species. It is distinguished from all species of *Alburnoides* in Turkey and adjacent watersheds by fewer total lateral-line scales (42–47, vs. 47–57, exceptfor *Alburnoides tzanevi*) and a longer caudal fin (length of the upper caudal-fin lobe 28–31% SL, vs. 21–28, see Table 1). Besides characters mentioned, *Alburnoides* cf. *smyrnae* differs from *Alburnoides tzanevi* by having a deeper body (28−30% SL, mean 28.6, vs. 24−27, mean 25.4).

*Alburnoides manyasensis* can be distinguishedfrom*Alburnoides* cf. *smyrnae* by having fewer branched anal-fin rays (10½–12½, vs. 12½–14½), a narrower interorbital distance (7–8% SL, mean 7.7, vs. 8–10, mean 8.9), the presence of a hump at nape in specimens larger than 60 mm SL (vs. absent), the ventral keel scaleless about 1/2 to 2/3 of its length (vs. almost entirely scaled) and the upper profile of head slightly convex at level of nostrils (Fig. 3) (vs. markedly convex, see Fig. 4).

The type locality of *Alburnoides bipunctatus tzanevi* is Rezovska River in Bulgaria, Black Sea drainage. We consider it to be a valid species because it can be easily distinguished from all species of *Alburnoides* in Turkey and adjacent area by a more slender body (the body depth at dorsal-fin origin about equal to the head length, vs. the body depth at dorsal-fin origin commonly greater than the head length), a considerably pointed snout (vs. slightly pointed or rounded). The detailed metric and meristic characters of *Alburnoides tzanevi* are given in Tables 1 and 3.

**Figure 5.**
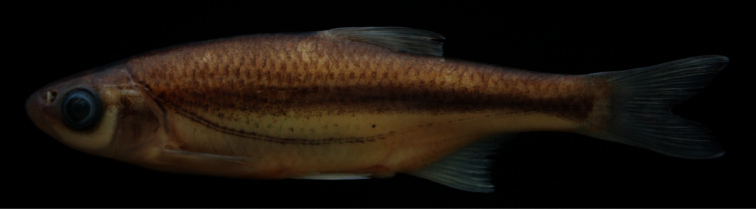
*Alburnoides tzanevi***;** Turkey: İstanbul Province: Terkos Stream, FFR 1066, female, 77 mm SL.

Besides the differences given above, *Alburnoides manyasensis* is distinguishedfrom *Alburnoides tzanevi* by the presence of a hump at nape in specimens larger than 60 mm SL (vs. absent) and a deeper caudal peduncle (11–12% SL, mean 11.5, vs. 9–11, mean 9.9). In *Alburnoides manyasensis*, pigmentation of the lateral line is slightly distinct in anterior part of the body but indistinct in posterior part of body (Fig. 3) and the snout length is markedly shorter than interorbital distance in contrast to *Alburnoides tzanevi* (Fig. 5) with the lateral line clearly distinct in both the anterior and posterior parts of the body and the snout length about equal to the interorbital distance.

**Figure 6.**
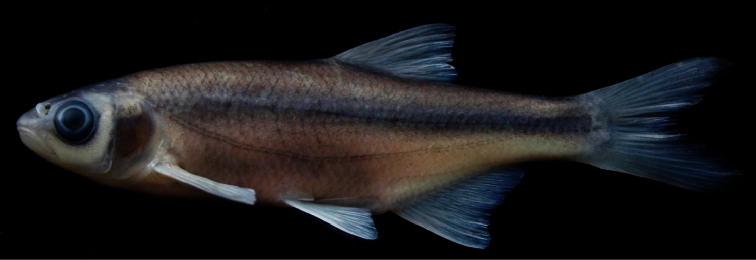
*Alburnoides fasciatus***,** Turkey: Artvin Province: Aralık Stream, Çoruh River drainage, FFR 1003, female, 75 mm SL.

*Alburnoides manyasensis* differs well from *Alburnoides fasciatus* by the presence of a hump at nape in specimens larger than 60 mm SL (vs. absent) and in having fewer branched anal-fin rays (10½–12½, vs. 13½–15½) and fewer predorsal vertebrae (13–14, mode 14, vs. 14–15, mode 15). It is further distinguishedfrom *Alburnoides fasciatus* by a more slender body (29–32% SL, mean 29.4, vs. 25–30, mean 27.8), a narrower head (head width at the anterior eye margin 8–10% SL, mean 8.8, vs. 10–11, mean 10.1) and a longer caudal peduncle (20–25% SL, mean 22.2, vs. 17–21, mean 19.1). In *Alburnoides manyasensis*, theeye diameter is longer than both the snout length and the interorbital width, while in *Alburnoides fasciatus* theeye diameter is smaller than both the snout length and the interorbital width (Table 2). In *Alburnoides manyasensis*, pigmentation of the lateral line is slightly distinct in the anterior part of the body but indistinct in the posterior part (Fig 3) in contrast to *Alburnoides fasciatus* (Fig. 6)with the lateral line clearly distinct in both the anterior and posterior parts of the body.

**Figure 7.**
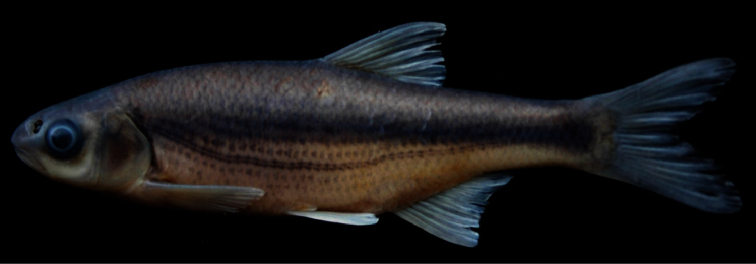
*Alburnoides eichwaldii*, Turkey: Ardahan Province: Hanak Stream, Kura River drainage, FFR 1047, female, 84 mm SL.

*Alburnoides manyasensis* is easily distinguished from *Alburnoides eichwaldii* by the presence a hump at nape in specimens larger than 60 mm SL (vs. absent), It further differs from *Alburnoides eichwaldii* by a more slender body (body depth at dorsal fin origin 29–32% SL, mean 29.4, vs. 25–30, mean 27.5). In *Alburnoides manyasensis*, theeye diameter is longer than the snout length and the interorbital width, while in *Alburnoides eichwaldii* the eye diameter is shorter than the snout length and the interorbital width (see Table 1, 2).In *Alburnoides manyasensis*, pigmentation of the lateral line is slightly distinct in the anterior part of the body but indistinct in the posterior part, while in *Alburnoides eichwaldii* (Fig. 7) the dots along the lateral line are distinct in both the anterior and posterior parts of the body.

**Figure 8.**
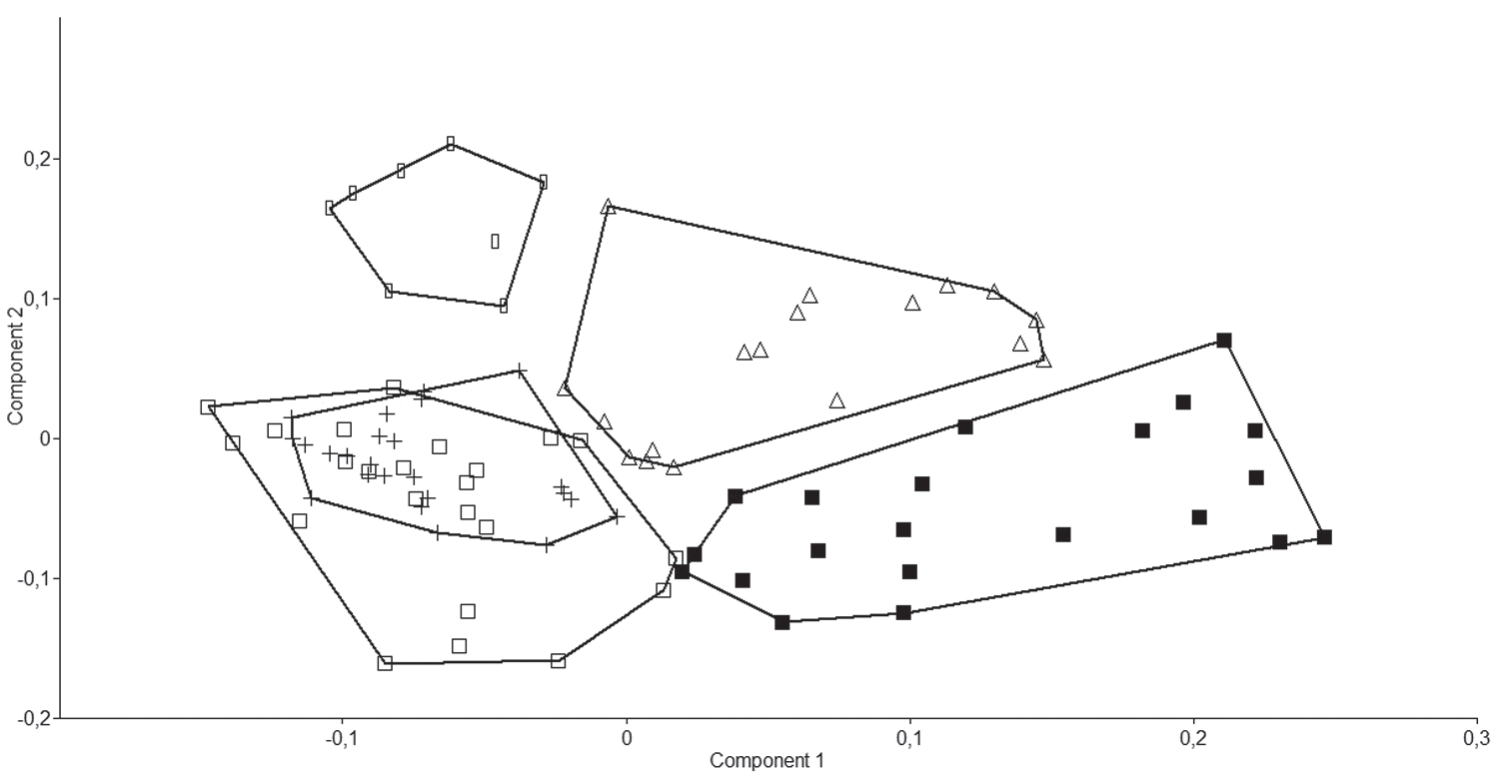
A scatter plot of the scores of the first two principal components (PC I, PC II) for 57 specimens of the five *Alburnoides* species (*Alburnoides manyasensis* (∆), *Alburnoides* cf. *smyrnae* ( █ ) *Alburnoides tzanevi* (■), *Alburnoides fasciatus* (+) and *Alburnoides eichwaldii* (□), based on 26 morphometric characters.

Five *Alburnoides* species from Turkey (*Alburnoides manyasensis*, *A*. cf. *smyrnae, A*. *tzanevi*, A. *fasciatus* and *Alburnoides eichwaldii*) were compared by Principal Component Analysis (PCA). The PCA was performed in using twenty–six morphometric characters of the five *Alburnoides* species. The PCA separated *Alburnoides manyasensis* from *Alburnoides* cf. *smyrnae, A*. *tzanevi*, *Alburnoides fasciatus* and *Alburnoides eichwaldii* (Fig. 8). Also,there was only marginal overlap between *Alburnoides fasciatus* and *Alburnoides eichwaldii* (Fig. 8). Variables loading on the first metric PC I–II are given in Table 4.

**Table 2.** Morphometric characters in *Alburnoides fasciatus* and *Alburnoides eichwaldii*.Mean values are given in parentheses.

	*Alburnoides fasciatus*, n=14		*Alburnoides fasciatus*, n=10		*Alburnoides eichwaldii*, n=15		*Alburnoides eichwaldii*, n=10	
Basin	Black Sea		Black Sea		Caspian Sea		Caspian Sea	
River or stream	Çoruh		İyidere		Aras		Kura	
	Range	SD	Range	SD	Range	SD	Range	SD
Standard length (mm)	60–77		52–67		55–107		55–87	
**In percents of standard length**								
Head length	25.5–27.5 (26.7)	0.55	24.5–28.3 (26.4)	1.57	24.3–27.7 (25.8)	1.16	25.8–27.6 (26.7)	0.68
Body depth at dorsal fin origin	25.7–29.3 (27.8)	1.14	25.4–29.7 (27.9)	1.97	25.0–29.6 (27.5)	1.28	25.7–30.1 (27.7)	1.90
Body depth at anal fin origin	22.6–25.6 (24.3)	0.91	22.3–24.2 (23.3)	0.81	21.7–24.4 (23.0)	0.86	21.1–25.3 (23.3)	1.22
Caudal peduncle depth	10.8–12.0 (11.4)	0.40	11.0–12.2 (11.5)	0.46	11.4–13.2 (12.1)	0.48	11.2–12.6 (11.9)	0.41
Predorsal length	53.0–55.6 (54.3)	0.76	52.8–56.1 (54.7)	0.99	50.6–54.6 (52.9)	1.09	52.8–56.3 (54.4)	1.25
Prepelvic length	45.9–49.2 (47.3)	0.99	45.8–50.0 (48.7)	1.27	45.6–48.8 (47.2)	0.85	46.3–50.1 (48.1)	1.34
Preanal length	64.2–68.4 (66.0)	1.25	65.2–69.1 (66.9)	1.19	63.3–66.2 (65.0)	0.83	64.1–69.2 (65.7)	1.60
Pectoral-fin origin to anal fin	40.4–44.3 (41.9)	1.25	41.7–45.9 (43.0)	1.26	39.4–44.2 (41.4)	1.37	39.5–46.1 (41.5)	2.13
Pectoral-fin origin to pelvic fin	20.9–23.9 (22.8)	0.66	22.6–26.3 (24.4)	1.08	21.5–26.6 (22.9)	1.29	22.1–26.2 (23.6)	1.30
Pelvic-fin origin to anal fin	16.7–20.5 (18.8)	1.09	17.9–20.6 (19.0)	0.78	16.9–20.1 (18.6)	0.98	16.2–21.0 (18.3)	1.60
Caudal peduncle length	18.1–20.6 (19.6)	0.68	16.9–20.0 (18.5)	0.96	19.6–23.3 (21.4)	0.97	19.2–21.3 (20.5)	0.75
Dorsal fin depth	23.0–25.7 (24.0)	0.74	22.3–25.7 (23.7)	1.04	20.1–25.2 (23.0)	1.44	21.4–26.0 (23.6)	1.39
Pectoral fin length	20.8–22.7 (21.6)	0.61	19.9–23.6 (21.9)	1.19	19.3–22.5 (20.7)	1.06	19.6–22.9 (21.5)	1.24
Pelvic fin length	16.6–18.3 (17.1)	0.47	15.9–19.4 (17.6)	1.18	15.7–18.5 (16.8)	0.92	15.7–18.8 (17.2)	0.94
Anal fin length	17.4–20.2 (18.2)	0.75	17.3–19.6 (18.1)	0.86	15.0–19.1 (16.8)	1.02	15.8–19.2 (17.4)	1.02
Upper caudal-fin lobe	21.9–26.6 (24.8)	0.14	23.9–27.6 (25.0)	1.21	20.5–27.3 (24.0)	2.25	22.2–27.0 (24.0)	1.32
Middle caudal-fin lobe	14.1–15.7 (14.8)	0.55	14.8–16.6 (15.8)	0.56	13.4–16.8 (15.1)	1.08	13.1–16.4 (15.2)	1.05
Head width_1_	9.5–10.7 (10.1)	0.29	9.5–10.5 (9.9)	0.39	9.2–10.7 (10.0)	0.47	9.1–10.2 (9.7)	0.31
Head width_2_	12.4–13.4 (13.0)	0.26	11.8–14.3 (12.9)	0.70	11.9–13.1 (12.6)	0.42	11.4–13.4 (12.6)	0.65
Head width_3_	13.3–14.6 (14.0)	0.42	12.7–14.1 (13.3)	0.47	13.1–15.0 (14.0)	0.66	12.4–14.7 (13.5)	0.67
Head depth_1_	14.4–16.3 (15.2)	0.48	13.7–15.9 (14.8)	0.71	13.3–15.0 (14.4)	0.45	14.3–15.5 (14.9)	0.31
Head depth_2_	18.8–20.8 (19.7)	0.45	17.5–20.3 (19.1)	0.85	18.0–20.6 (19.4)	0.66	18.0–20.2 (19.4)	0.63
Eye diameter	7.0–8.1 (7.6)	0.33	7.3–8.0 (7.7)	0.23	5.9–7.3 (6.7)	0.47	6.5–8.1 (7.4)	0.61
Snout length	7.5–8.1 (7.7)	0.19	7.7–8.2 (7.8)	0.18	7.2–8.5 (7.8)	0.38	7.0–8.0 (7.6)	0.38
Interorbital width	8.7–9.8 (9.2)	0.32	8.0–9.4 (8.9)	0.44	7.8–8.8 (8.4)	0.31	7.4–9.5 (8.3)	0.59
Snout width at nostrils	9.1–10.6 (9.9)	0.39	9.4–10.8 (10.2)	0.55	9.6–10.7 (10.2)	0.29	10.0–11.3 (10.5)	0.40

**Table 3.** Frequency of occurrence of meristic characters in five *Alburnoides* species distributed in Turkey.

	**Lateral-line scales**																
	42	43	44	45	46	47	48	49	50	51	52	53	54	55	56	57	Mean
*Alburnoides manyasensis*, n=25	–	–	–	–	–	2	3	1	3	5	5	4	–	2	–	–	50.9
*Alburnoides cf smyrnae*, n=8	1	3	1	1	1	1	–	–	–	–	–	–	–	–	–	–	44.3
*Alburnoides tzanevi*, n=17	–	1	1	–	3	4	1	–	4	1	2	–	–	–	–	–	48
*Alburnoides fasciatus*, n= 30	–	–	–	–	–	1	4	8	7	3	6	–	1	–	–	–	50
*Alburnoides eichwaldii*, n= 38	–	–	–	–	–	–	5	3	2	5	8	8	2	3	–	1	51.7
	**Scales above lateral line**					**Scales below lateral line**				**Branched anal-fin rays**							
	9	10	11	12	Mean	4	5	6	Mean	10	11	12	13	14	15	Mean	
*Alburnoides manyasensis*, n=25	1	8	14	2	10.7	5	20	–	4.2	2	9	14	–	–	–	11.5	
*Alburnoides cf smyrnae*, n=8	3	5	–	–	9.6	8	–	–	8	–	–	1	4	2	–	13	
*Alburnoides tzanevi*, n=17	16	1	–	–	9.1	17	–	–	4	–	6	11	–	–	–	11.6	
*Alburnoides fasciatus*, n=30	1	24	5	–	10.1	3	27	–	4.9	–	–	–	16	12	2	13.5	
*Alburnoides eichwaldii*, n=38	11	23	4	–	9.8	4	32	2	4.9	–	3	20	13	2	–	12.4	
	**Total vertebrae**					**Abdominal vertebrae**				**Caudal vertebrae**							
	40	41	42	43	Mean	20	21	22	Mean	19	20	21	22	Mean			
*Alburnoides manyasensis*, n=10	3	5	2	–	40.9	1	8	1	21	2	6	2	–	20			
*Alburnoides* cf *smyrnae*, n=8	–	6	2	–	41.3	3	5	–	20.6	–	6	2	–	20.3			
*Alburnoides tzanevi*, n=9	1	3	5	–	41.4	2	5	2	21	–	6	2	1	20.4			
*Alburnoides fasciatus*, n=10	–	4	5	1	41.7	–	5	5	21.5	2	4	4	–	20.2			
*Alburnoides eichwaldii*, n=10	–	–	10	–	42	–	10	–	21	–	–	10	–	21			

**Table:** **Table 4.** Character loading on principal components I–II for 26 measurements taken on 96 specimens of five *Alburnoides* species (*Alburnoides manyasensis*, *Alburnoides* cf. *smyrnae*, *Alburnoides tzanevi*, *Alburnoides fasciatus* and *Alburnoides eichwaldii*).

Morphometric features (% SL)	PC I	PC II
Head length	–0.008	0.064
Body depth at dorsal–fin origin	0.009	0.122
Body depth at anal–fin origin	–0.056	0.118
Caudal peduncle depth	–0.175	0.142
Head width_1_ (ant. margin of eye)	–0.358	0.066
Head width_2_ (post. margin of eye)	–0.055	0.177
Head width_3_ (at opercle)	–0.135	0.173
Head depth_1_ at interorbital region	–0.161	0.079
Head depth_2_ (at nape)	–0.098	0.112
Eye diameter	0.353	0.311
Snout length	–0.322	0.165
Interorbital width	–0.355	0.275
Snout width at nostrils	–0.554	–0.158
Predorsal length	0.015	0.047
Prepelvic length	–0.018	0.000
Preanal length	–0.032	–0.031
Pectoral–fin origin to anal fin	–0.036	–0.098
Pectoral–fin origin to pelvic fin	–0.021	–0.080
Pelvic–fin origin to anal fin	–0.027	–0.161
Caudal-peduncle length	0.133	0.107
Dorsal–fin depth	0.144	0.356
Pectoral–fin length	0.035	0.248
Pelvic–fin length	0.003	0.224
Anal–fin length	0.182	0.245
Upper caudal–fin lobe	–0.068	0.213
Middle caudal–fin lobe	0.022	0.375

## Comparative material

*Alburnoides eichwaldii*: FFR 1013, 3, 60–82 mm SL; Turkey: Ardahan Prov.: Aşıkzülal Stream, Kura River drainage; D. Turan & R. Buyurucu, 02 Sep. 2006. − FFR 1019, 2, 75–87 mm SL; Turkey: Ardahan Prov.: Susuz Stream, Aras River, Kura River drainage; D. Turan & R. Buyurucu, 23 June 2006. – FFR 1022, 112, 39–88 mm SL; Turkey: Ardahan Prov.: Kura River drainage; D. Turan & R. Buyurucu, 20 Sep. 2004. – FFR 1038, 6, 48–63 mm SL; Turkey: Kars Prov.: Selim Stream, Aras River, Kura River drainage; D. Turan & R. Buyurucu, 04 July 2004. – FFR 1039, 8, 47–87 mm SL; Turkey: Kars Prov.: Kars Stream, Aras River, Kura River drainage; D. Turan & R. Buyurucu, 19 Aug. 2007. – FFR 1047, 38, 50–107 mm SL; Turkey: Ardahan: Hanak Stream, Kura River drainage; D. Turan & R. Buyurucu, 12 June 2005. – FFR 1063, 67, 57–106 mm SL; Turkey: Kars Prov.: Boyalı Stream, Aras River, Kura River drainage; D. Turan & R. Buyurucu, 05 July 2007. – FFR 1071, 29, 31–103 mm SL; Turkey: Kars Prov.: Sarıkamış Stream, Aras River, Kura River drainage; D. Turan & R. Buyurucu, 03 Sep. 2006.– FFR 1082, 24, 33–94 mm SL; Turkey: Kars Prov.: Kızılçubuk Stream, Aras River, Kura River drainage; D. Turan & R. Buyurucu, 03 Sep. 2006. – FFR 1084, 7, 34–75 mm SL; Turkey: Ardahan: Göle Stream, Kura River drainage; D. Turan & R. Buyurucu, 06 Sep. 2006. – FFR 1085, 32, 41–89 mm SL; Turkey: Ardahan Prov.: Çıldır Lake, Aras River, Kura River drainage; D. Turan, C. Kaya & E. Doğan, 14 July 2012. – FFR 1087, 2, 63–78 mm SL; Turkey: Iğdır Prov.: Perçekkale Stream, Aras River, Kura River drainage; D. Turan, C. Kaya & E. Doğan, 16 July 2012. – FFR 1088, 37, 65–113 mm SL; Turkey: Kars Prov.: Selim Stream, Aras River, Kura River drainage; D. Turan, C. Kaya & E. Doğan, 15 July 2012. – FFR 1089, 7, 82–112 mm SL; Turkey: Ardahan Prov.: Yalnızçam Stream, Kura River drainage; D. Turan, C. Kaya & E. Doğan, 19 July 2012. – FFR 1090, 10, 39–78 mm SL; Turkey: Iğdır Prov.: Aras River, Kura River drainage; D. Turan, C. Kaya & E. Doğan, 17 July 2012. – FFR 1091, 18, 61–96 mm SL; Turkey: Ardahan Prov.: Göle Stream, Kura River drainage; D. Turan, C. Kaya & E. Doğan, 14 July 2012. – FFR 1092, 14, 52–91 mm SL; Turkey: Ardahan Prov.: Hanak Stream, Kura River drainage; D. Turan, C. Kaya & E. Doğan, 14 July 2012. – FFR 1093, 19, 63–85 mm SL; Turkey: Kars Prov.: Digor Stream, Aras River, Kura River drainage; D. Turan, C. Kaya & E. Doğan, 17 July 2012.

*Alburnoides fasciatus*: FFR 1000, 16, 28–57 mm SL; Turkey: Artvin Prov.: Hopa Stream; D. Turan, C. Kaya & E. Doğan, 11 Nov. 2011. – FFR 1003, 30, 62–73 mm SL; Turkey: Artvin Prov.: Aralık Stream, Çoruh River drainage; D. Turan, C. Kaya & E. Doğan, 15 July 2011. – FFR 1004, 6, 52–81 mm SL; Turkey: Artvin Prov.: Hopa Stream; D. Turan & R. Buyurucu, 23 June 2007. – FFR 1006, 9, 59–91 mm SL; Turkey: Rize Prov.: İyidere Stream; D. Turan & R. Buyurucu, 3 Jan. 2007. – FFR 1007, 3, 46–78 mm SL; Turkey: Rize Prov.: İyidere Stream; D. Turan & R. Buyurucu, 3 May 2009. – FFR 1008, 2, 55–64 mm SL; Turkey: Giresun Prov.: Aksu Stream; D. Turan & R. Buyurucu, 6 Sep. 2004. –FFR 1009, 43, 37–98 mm SL; Turkey: Rize Prov.: İyidere Stream; D. Turan & R. Buyurucu, 19 May 2008. – FFR 1011, 16, 67–87 mm SL; Turkey: Rize Prov.: Büyükçay Stream; D. Turan & R. Buyurucu, 10 Sep. 2004. – FFR 1024, 2, 67–72 mm SL; Turkey: Rize Prov.: Güneysu Stream; D. Turan & R. Buyurucu, 27 July 2006. – FFR 1046, 60, 41−88 mm SL; Turkey: Artvin Prov.: Aralık Stream, Çoruh River drainage; D. Turan & R. Buyurucu, 20 July 2007. – FFR 1081, 41, 57–89 mm SL; Turkey: Rize Prov.: Büyükçay Stream; D. Turan & R. Buyurucu, 29 July 2006.

*Alburnoides* cf. *smyrnae*: FFR 1110, 8, 58−77 mm SL; Turkey: Uşak Prov.: Banaz Stream, Menderes River drainage; S. S. Güçlü, 7 Sep. 2012.

*Alburnoides tzanevi*: FFR 1049, 3, 48−94 mm SL; Turkey: İstanbul Prov.: Istranca Stream; D. Turan & R. Buyurucu, 15 Aug. 2005. – FFR 1052, 9, 33−80 mm SL; Turkey: İstanbul Prov.: Karamandere Stream; D. Turan & R. Buyurucu, 10 July 2007. – FFR 1066, 10, 63–81 mm SL; Turkey: İstanbul Prov.: Terkos Stream; D. Turan & R. Buyurucu, 10 July 2007. – FFR 1068, 17, 63-96 mm SL; Turkey: İstanbul Prov.: Istranca Stream; D. Turan & R. Buyurucu, 15 Aug. 2005.

## Supplementary Material

XML Treatment for Alburnoides manyasensis
